# The effectiveness of a health education intervention to reduce anxiety in quarantined COVID-19 patients: a randomized controlled trial

**DOI:** 10.1186/s12889-023-16104-w

**Published:** 2023-06-20

**Authors:** Imen Zemni, Amel Gara, Hadhba Nasraoui, Meriem Kacem, Amani Maatouk, Oumeyma Trimeche, Hela Abroug, Manel Ben Fredj, Cyrine Bennasrallah, Wafa Dhouib, Ines Bouanene, Asma Sriha Belguith

**Affiliations:** 1 Department of Epidemiology and Preventive Medicine, Fattouma Bourguiba University Hospital, Monastir, Tunisia; 2 Department of Epidemiology, Faculty of Medicine of Monastir, University of Monastir, Monastir, Tunisia; 3 Technology and Medical Imaging Research Laboratory - LTIM - LR12ES06, University of Monastir, Monastir, Tunisia; 4 Department of Emergency Medicine, Taher Sfar University Hospital, Mahdia, Tunisia; 5 Department of Endocrinology, Fattouma Bourguiba University Hospital, Monastir, Tunisia

**Keywords:** Health education, COVID-19, Quarantine, Anxiety, Pandemic

## Abstract

**Introduction:**

The COVID-19 pandemic is regarded as a serious public health concern that boosts levels of stress and anxiety which could be explained by several reasons, including social isolation. In this regard, we aimed to assess the impact of health education on the anxiety level of COVID-19 patients during the isolation period.

**Methods:**

This is a randomized controlled trial conducted between February 2021 and June 2021. Patients tested positive for Covid-19 with mild to moderate forms were randomized to Education (n = 267) or control (n = 269). The education group received a phone health education session on day 1 (D1) following the diagnosis. The three components of the health education intervention were an explanation of the coronavirus disease, what to do in the event of complications, and the recommended preventive measures. The two groups received a telephone evaluation of their Hospital Anxiety and Depression scores on D1 and day seven D7 following the positive diagnosis. The primary outcome was the rate of anxiety reduction in each group on D7 based on a HAD-A score ≥ 8. Secondary outcomes were the rate of anxiety reduction on D7 based on a HAD-A score ≥ 11, the percentage of people complying with isolation and the scores of adherences to preventive measures during the isolation in each group.

**Results:**

Hundred and ninety-six patients in the intervention group and 206 patients in the control group completed the study. The sociodemographic, clinical, and initial anxiety level features of the intervention and control groups were comparable at baseline (p ≥ 0.05). On D7, the education group’s anxiety level (HAD-A ≥ 8) decreased from 26 to 16.3% (p = 0.013) while in the control group it increased from 19.4 to 22.8% (p = 0.37). Thus, the percentage change in anxiety between D1 and D7 (delta D7 – D1) was − 9.7% in the Education group and + 3.4% in the Control group. Using the HAD-A ≥ 11 thresholds, the percentage of anxiety decreased from 15.3 to 11.2% (p = 0.26) between D1 and D7, while it increased in the control group from 9.7 to 15.7% (p = 0.045). Thus, the education group’s change in anxiety (delta D7 - D1) was − 4.1%, while the control group’s change was + 6%.

**Conclusion:**

During an outbreak, providing health education to quarantined patients may be beneficial to reduce the psychological impact of the disease.

**Trial registration number:**

ClinicalTrials.gov Identifier: NCT05715593, retrospectively registered on 8/02/2023 https://clinicaltrials.gov/ct2/results?term=NCT05715593&Search=Search.

**Supplementary Information:**

The online version contains supplementary material available at 10.1186/s12889-023-16104-w.

## Introduction

The world has been facing an unprecedented major health crisis due to the coronavirus outbreak which has caused around 7 million death globally [1]. In Tunisia, almost 450,000 confirmed cases of COVID-19 and 20,000 related deaths were detected until June 2021 [2].

This pandemic led to a considerable psychological and behavioral change in daily lives [3, 4]. Indeed, since the beginning of SARS-CoV2 outbreak, many preventive measures have been, worldwide, imposed including disinfection, lockdown and home quarantine for infected people.

The recommendations called for isolation of patients who are infected, exposed, or have symptoms suggestive of Covid-19 which further increased levels of stress and anxiety [5]. According to the literature, being socially isolated, can lead to the development of anxiety-depressive syndromes, severe psychological distress and a great sense of loneliness [6–8]. The impact of lockdown and quarantine on mental health has been widely documented in several studies [9–11].

Furthermore, many other reasons could increase the likelihood of anxiety and other mental health problems, such as the lack of knowledge about the disease, the daily increases in the number of confirmed cases and deaths, and the fear of infecting family members and putting their lives in danger [9].

It has long been established that health education is an effective strategy for reducing disease’s consequences and impact. By providing information, and support, health education has been shown to reduce psychological impact of the disease [12, 13].

That is why, education of Covid-19 patients during quarantine in order to help them coping with their health status, could prevent psychological effects including anxiety [7]. Many publications highlighted the impact of health education in reducing mental health problems during COVID-19 pandemic in general and specific population (students, mothers…) [14–17]. However, most of these publications was observational studies. Only few experimental studies have focused on mental health during COVID-19 [18–20]. According to our knowledge, there is no published studies dealing with the psychological effect of health education in quarantined Covid-19 patients.

Within this context, the aim of our study was to assess the impact of health education on the anxiety level of Covid-19 patients during quarantine.

## Methods

### Trial design

This is a randomized controlled trial with two parallel groups (Education and Control), assessing the effectiveness of a health education intervention on anxiety levels in isolated patients with COVID-19.

### Participants

Participants were recruited from the Covid-19 testing unit at the Fattouma Bourguiba hospital of Monastir between February 2021 and June 2021. Inclusion criteria were: (1) Patients diagnosed with Covid-19 after positive PCR or positive antigen rapid test, (2) over 18 years of age and (3) who did not require hospitalization.

Patients not reachable at the first day phone call of the diagnosis, patients who declined to participate, and patients unable to answer the telephone questionnaire due to their cognitive impairment (dementia….) or diagnosed with a mental health problem were excluded.

### Intervention

Since the beginning of the pandemic, all patients diagnosed with COVID-19 have received a call from the Department of Preventive Medicine of Fattouma Bourguiba university hospital one day after the diagnosis is confirmed, to identify individuals who may have been in close contact with them (Contact tracing). In addition to the usual contact tracing, the intervention group received a telephone health education session on the first day of confirmation of the diagnosis. Calls were delivered by medical residents who were trained in communication skills and on intervention delivery prior to initiation of the study. Each call lasted approximately 10 to 15 min. The educators targeted three axes of messages: (Axe 1) Explanation of the Coronavirus disease, (Axe 2) Self-monitoring and what to do in case of complications, (Axe 3) Preventive measures to respect (Box 1). During the phone call, each educator had a checklist of the detailed educational messages written in Tunisian dialect in front of him on a tablet to guide him to normalize the educational intervention. To avoid forgetting certain messages, the educator had to check each transmitted message.

**Table:** Box 1: Topics of the educational messages

**Topic 1 Explanation of the Coronavirus disease**
• Symptoms are almost similar to those of the flu virus. • The duration of symptoms does not exceed the week generally. • There is no reason to panic especially for those who are immunocompetent • The virus can cause some complications for some vulnerable categories of people. • In case of chronic disease or old age, it is recommended to have a close follow-up but not to the point of panicking.
**Topic 2 Self-monitoring and what to do in case of complications**
• How to self-monitor at home (symptoms, temperature, and saturation) • When to consult in the emergency department or to call the Urgent Medical Assistance Service (In case of respiratory difficulty, acute dyspnea, chest pain or hypoxia)
**Topic 3 Preventive measures to respect**
Preventive measures should always be respected, especially for those living with vulnerable people: • To be self-isolated in his/her room • To wash hands frequently • Not to share personal tools with others (towels, water bottle…) • To use a personal toilet block, if possible. Otherwise, thoroughly clean with bleach before leaving. • To wear the mask every time when leaving his/her room • To air the room regularly • To put the waste in double bags in front of the room • To keep a distance of at least one meter from anyone when leaving room

### Outcomes

The primary outcome of this trial was the rate of anxiety reduction in each group on day seven of the date of diagnosis. Anxiety levels were defined according to the Arabic version of the Hospital Anxiety and Depression (HAD) scale [21]. The (HAD-A) sub-scale is used to screen for anxiety disorders and is composed of 7 items. The items were measured on a four-point scale (from 0 to 3). The resultant overall score ranged from 0 to 21: (0–7 (No anxiety) and ≥ 8: (anxiety)). Anxiety levels are as follows: 8–10 (Mild); 11–15 (Moderate); 16–21 (Severe)).

The primary outcome was calculated based on a HAD-A score ≥ 8.

The Secondary outcomes of this study were: (1) the rate of anxiety reduction in each group on day seven of the date of diagnosis based on a HAD-A score ≥ 11, (2) the percentage of people complying with quarantine in each group (assessed based on the frequency of quarantine break-up during the isolation period for reasons other than seeking care), (3) The scores of adherence to preventive measures during the isolation in each group (evaluated by a Likert scale from 0 = Never to 4 = Always). The assessed preventive measures were the wearing of masks, washing hands, sharing objects with others, distancing, room aeration, waste management and disinfection of the sanitary block.

### Sample size

To calculate the sample size we used the online software BiostaTGV with a power of 80%, a 2-sided 5% level of significance, 29% prevalence of anxiety in Covid-19 patients [22] and 15% as a target prevalence. The minimum number of patients required was 268 subjects (134 subjects in each group). Under the assumption that at least 25% of the participants would drop out, we required an adjusted sample size of 358 patients (179 in each group at least) using the following formula: N = n / (1-(z/100)). N is the final adjusted sample size, n is the calculated sample size and z% is the expected dropout rate [23].

### Randomization

After patients’ eligibility was confirmed and the informed consents were obtained, enrolled patients were randomly assigned to education group and control group in a 1:1 ratio using a computer-generated sequence.

### Data collection

The two groups were called by telephone on Day 1 and Day 7 of the positive diagnosis and were asked to respond to a telephone questionnaire. The data collected on Day 1 were: age, sex, comorbidities (hypertension, diabetes, dyslipidemia, cardiac pathology, respiratory pathology, renal pathology, psychiatric pathology, immunodeficiency……), smoking status, and the baseline HAD (Hospital Anxiety and Depression Scale) score. Those collected on Day 7 were: HAD score, symptoms developed (fever, dyspnea, cough, anosmia, ageusia, odynophagia, asthenia, headache, myalgia aches, rhinitis, abdominal pain, chest pain, nausea, vomiting, diarrhea, confusion), the development of complications (hypoxia, pneumonia, respiratory failure, pulmonary embolism, panic attacks, insomnia…), compliance with preventive measures, compliance with quarantine and secondary hospitalization.

### Statistical analysis

Statistical analysis was performed using SPSS version 20.0. Categorical variables were summarized using numbers and percentages. Quantitative variables were expressed as mean ± standard deviations (SD) or as median (interquartile ranges, IQR) after testing for normality. The Student’s t-test for independent samples, Mann Whitney, Chi-square and Fisher’s exact t-tests were used to compare the baseline characteristics of the two study groups. For each group, the Wilcoxon test was used to compare the HAD-A score on Day1 and Day 7 and the McNemar test to compare the prevalence of anxiety on Day 1 and Day 7. The DELTA difference between the scores (HAD-A _D7_ - HAD-A _D1_) was compared between the two groups using the Man Whitney test. Results were considered significant at a threshold of p < 0.05.

### Ethical considerations

All procedures were performed under the tenets of the 1964 Helsinki Declaration. Informed consent was obtained from every patient after explaining the aim and the procedure of the study. Participants were given the opportunity to ask questions and decide whether to participate. The ethics committee of the faculty of medicine of Monastir approved the study. This trial was registered with clinical trial.gov (Trial registration Number: NCT05715593).

## Results

**Fig. 1:**
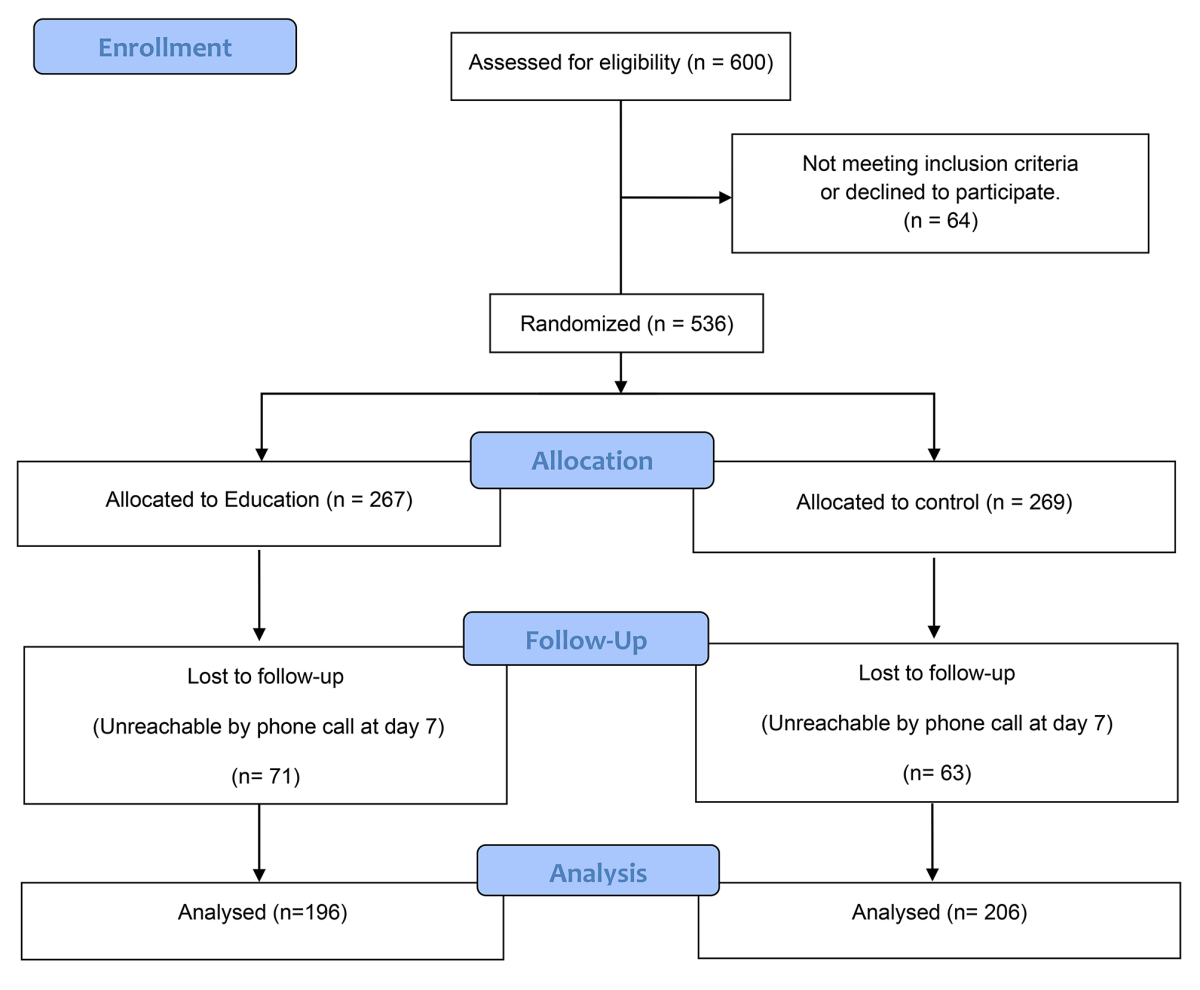
Flow Diagram

A total of 3174 patients were diagnosed with Covid-19 in the coronavirus testing unit of Monastir university hospital between February and June 2021.From them, 600 patients were randomly selected and assessed for eligibility. Sixty-four patients were not included for failing to meet one of the inclusion criteria. Among the 536 successful phone calls on day one ,134 patients did not respond to our phone calls on day seven even though we tried to reach them for at least three times on different days. Thus, we ended up with 402 patients (196 in the education group and 206 in the control group) as shown in the flowchart in Fig. 1.

### General and clinical characteristics of the two study groups

The average age of patients was 40.6 ± 15.87 years in the education group and 41.6 ± 14.35 years in the control group. The sex ratio was 0.67 in the education group and 0.54 in the control group. The frequency of comorbidities (hypertension, diabetes, dyslipidemia…) was comparable between the two groups. The general and clinical characteristics of the two groups are summarized in Table 1. Regarding the psychological profile of our population, median anxiety and depression scores assessed using the HAD scale on day 1 of the diagnosis were also comparable between the two groups (Table 2). The most common clinical symptoms in both groups were cough, asthenia, headache, anosmia, and fever with no statistically significant differences. The prevalence of complications in both groups was also similar (Table 1).

**Table 1:** General and clinical characteristics of the study population

	Education Group (n = 196)	Control Group (n = 206)		p
**Age (mean ± SD)**	40.6 ± 15.87	41.61 ± 14.35	0.5	
**Gender**				
Male	79 (40.3%)	73(35.41%)	0.31	
Female	117 (59.7%)	133(64.6%)		
**Comorbidities**				
Diabetes	20 [10.2%]	16 [7.8%]	0.39	
Hypertension	21 [10.7%]	19 [9.2%]	0.61	
Dyslipidemia	17 [9%]	20 [10.2%]	0.68	
Immunodeficiency	2 [1%]	2 [1%]	0.99*	
Respiratory disease	8 [4.1%]	10 [4.9%]	0.7	
**Smoking**	32 [17.4%]	26 [13.8%]	0.34	
**BMI (mean ± SD)**	27.1 ± 4.87	26.81 ± 4.49	0.53	
**Quarantine**				
Containment center	6 [3.3%]	11 [5.8%]	0.41*	
At home (home alone)	35 [19.2%]	41 [21.5%]		
At home (with family)	141 [77.5%]	139 [72.8%]		
**Symptoms developed**				
Fever	44 (23.9%)	49 (25.8%)	0.67	
Cough	72 (39,1%)	76 ( 40%)	0.86	
Dyspnea	26 (14.1%)	28 (14.7%)	0.86	
Anosmia	61 (33.2%)	61 (32.1%)	0.82	
Agueusia	48 (26.1%)	39 (2O.5%)	0.2	
Asthenia	70 (38%)	76 (40%)	0.69	
Headaches	69 (37.7%)	59 (31.1%)	0.17	
Rhinitis	18 (9.8%)	24 (12.7%)	0.37	
Odynophagia	11 (6%)	18 (9.5%)	0.2	
Myalgia	20 (10.9%)	28 (14.7%)	0.26	
Diarrhea	38 (20.7%)	44 (23.2%)	0.55	
Chest pain	21 (11.4%)	17 (9%)	0.44	
Arthralgia	3 (1.7%)	4 (2.3%)	0.72*	
Abdominal pain	5 (2.9%)	6 (3.5%)	0.74	
**Complications developed**				
Hypoxia	11 [5.8%]	14 [7.1%]	0.58	
Secondary hospitalization	6 [3.3%]	2 [1.1%]	0.17*	
Panic attack	11 [8.4%]	9 [6.9%]	0.64	
Insomnia	17 [12.5%]	15 [10.9%]	O.67	
Pneumonia	2 [1.5%]	5 [3.7%]	0.28*	
Pulmonary embolism	1 [0.8%]	1 [0.8%]	0.99*	
Respiratory failure	4 [3.1%]	3 [2.2%]	0.71*	

Comparison of percentages with Fisher’s test

**Table 2:** Comparison of the psychological profile of the two groups on Day 1 of diagnosis confirmation

	Education Groupe (n = 196)	Control Groupe (n = 206)	p
**Initial HAD-score**, Median [IIQ]	7[3;15.75]	6.5[2;15]	0.37
**Initial anxiety-score**, Median [IIQ]	3[1;8]	2[0;6]	0.16
**initial depression-score**, Median [IIQ]	4[2;9]	4[1;8]	0.71
**Anxiety (HAD-A) ≥ 8**			
Yes	51[26%]	40[19.4%]	0.11
No	145[74%]	166[80.6%]	
**Anxiety (HAD-A) ≥ 11**			
Yes	30[15.3%]	20[9.7%]	0.08
No	166 [84.7%]	186[90.3%]	

### Impact of education on levels of anxiety among participants

**Fig. 2:**
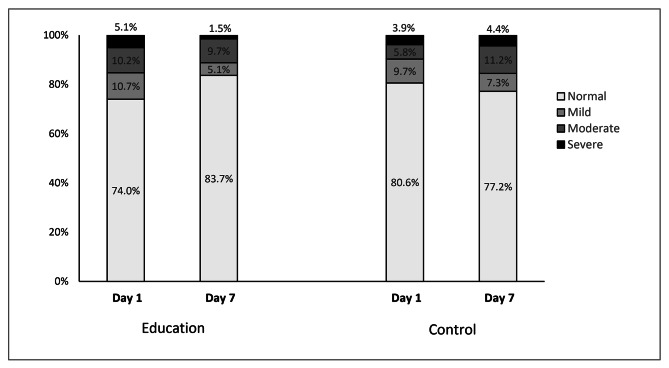
Comparison between baseline and final anxiety prevalence according to the Anxiety subscale of the HAD Scale among the two study groups

**Fig. 3:**
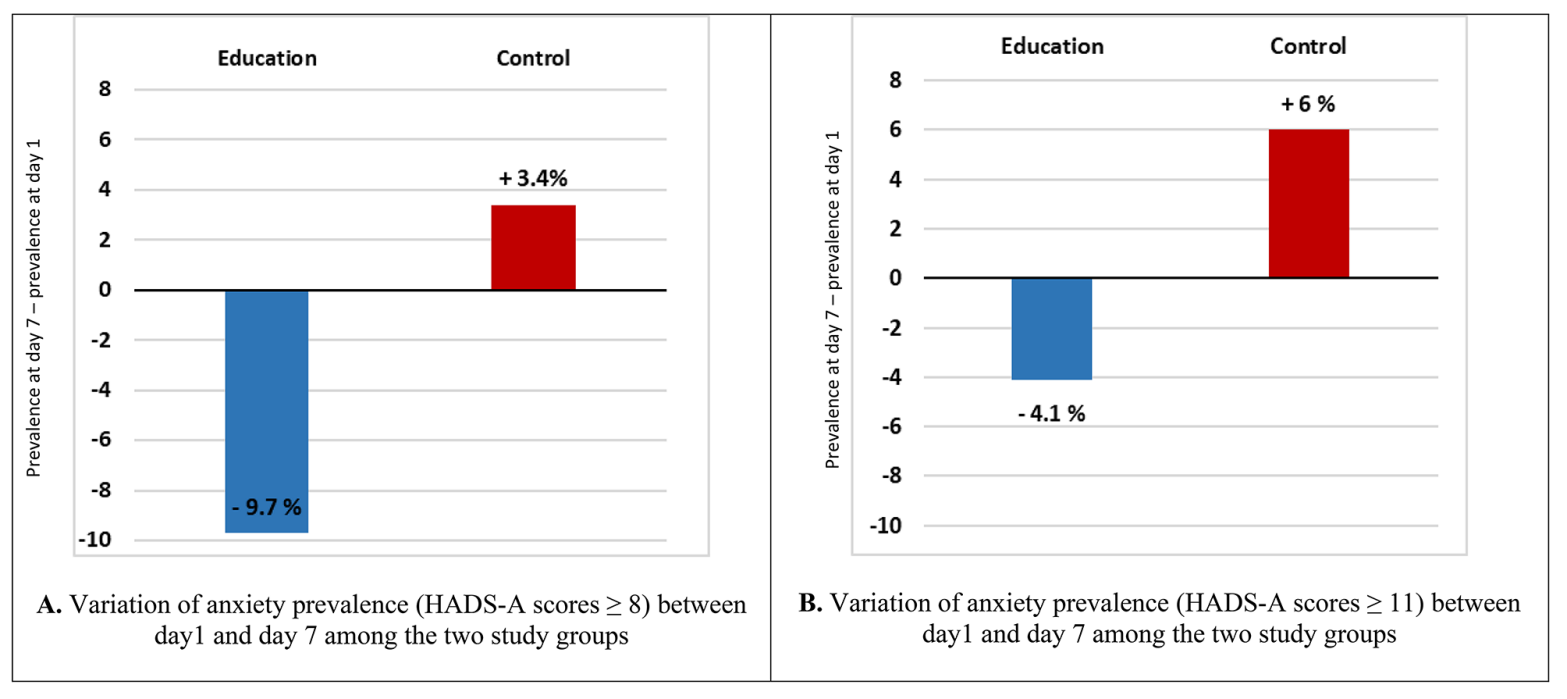
Variation of anxiety prevalence between day1 and day7 among the two study groups

Figure 2 shows a reduction in the percentage of anxiety (HAD-A ≥ 8) in the education group from 26 to 16.3% (p = 0.013) while in the control group it increased from 19.4 to 22.8% (p = 0.37). Thus, the percentage change in anxiety between day 1 and day 7 (delta Day7 – Day1) was − 9.7% in the education group and + 3.4% in the control group (Fig. 3.A).

Using the HAD-A ≥ 11 threshold, the percentage of anxiety decreased from 15.3 to 11.2% (p = 0.26) between Day 1 and Day 7 in the education group versus an increase in this percentage in the control group from 9.7 to 15.7% (p = 0.045) (Fig. 2). Thus, the education group’s change in anxiety (delta D7 - D1) was − 4.1%, while the control group’s change was + 6% (Fig. 3.B).

**Fig. 4:**
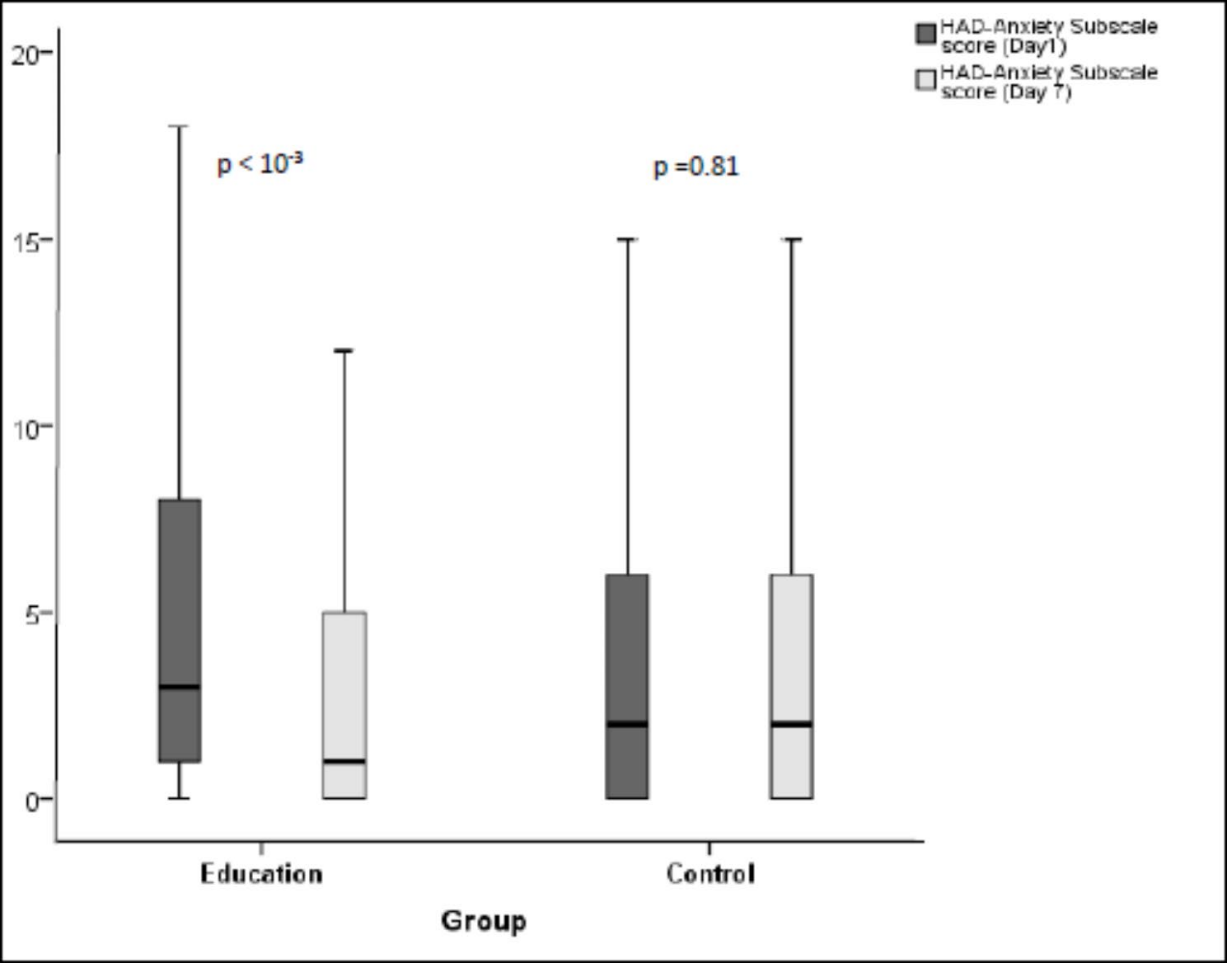
Comparison between distribution of baseline and final HADS-A scores among the two study groups

**Fig. 5:**
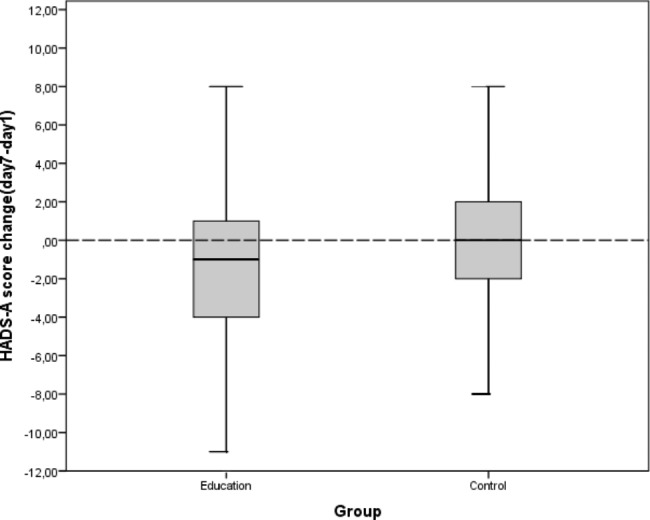
HADS-A scores difference (day7-day1) among the two groups

In the education group, the median anxiety score (HAD-A) was 3[1;8] on Day 1 and decreased to 1[0; 5] on Day 7 (p < 10^− 3^) while in the control group it increased from 2 [0;6] to 2 [0;6.25] with no statistically significant difference (p = 0.81) (Fig. 4). The change in score (HAD-A) between Day 1 and Day 7 (delta D7-D1) was significantly greater in the education group than in the control group with median delta values of -1 [− 4; +1] and 0 [-2; +2] respectively (p = 0.011) (Fig. 5).

### Impact of education on compliance with preventive measures during quarantine

Comparison of the mean scores of items assessing compliance with preventive measures during quarantine did not show a statistically significant difference (Table 3).

**Table 3:** Comparison of the average scores of the items evaluating the degree of hygiene compliance

	Education Group (mean ± SD)	Control group (mean ± SD)	P
I wear the mask every time I leave my room	3.06 ± 1.43	2.98 ± 1.47	0.6
I wash my hands or apply hydroalcoholic gel frequently	3.45 ± 0.98	3.50 ± 0.95	0.63
I do not share my objects (towel, linen, etc.) with others	3.48 ± 1.08	3.59 ± 1.03	0.29
I eat alone and I do not share foods with others	3.40 ± 1.21	3.30 ± 1.32	0.44
I make sure to clean the toilet block with bleach before going out	3.49 ± 1.02	3.52 ± 1.04	0.79
I make sure to ventilate my room daily	3.61 ± 0.91	3.54 ± 1.00	0.46
I put my waste in a double bag in front of the bedroom	3.00 ± 1.35	2.87 ± 1.42	0.35
I keep a distance of at least one meter from anyone when I leave my room	3.54 ± 3.17	3.21 ± 1.29	0.16

### Impact of education on quarantine compliance

The proportion of people who interrupted the quarantine during the isolation period for unjustified reasons was 17.6% in the education group and 15.2% in the control group with no statistically significant differences (p = 0.52).

## Discussion

Several studies support the significant relationship between quarantine and poor mental health during the COVID-19 pandemic. It was shown that the longer the quarantine lasted, the higher the anxiety, depression and stress levels are. Many reasons could explain this poor mental health outcomes such as the lack of knowledge about the disease, the daily increase in the number of confirmed cases and deaths and the fear of infecting family members and putting their lives in danger [9].

The current findings suggested that health education may play a conspicuous role in alleviating the psychological burden of isolated patients with Covid 19. In light of this, several authors have emphasized the importance of setting up a follow-up (telemedicine, tele-psychiatry) in order to minimize the mental impact of covid, particularly during the isolation period [24, 25].

Therefore, researchers in our study developed an education program that focused on explaining the nature of the disease, how to act if complications and the preventive measures to adopt during the isolation period and evaluated the effectiveness of this program in reducing anxiety levels.

According to our findings, the prevalence of isolated patients with Covid 19 suffering from anxiety with (HAD_A score ≥ 8) was 22.6%. The two groups of this trial were characterized by comparable general data, levels of anxiety and depression at baseline. Interestingly, patients in the education group experienced significant improvements in their self-reported anxiety symptoms at one week follow-up.

Our study aligns with existing literature and supports the understanding that individuals with extensive knowledge about COVID-19 tend to experience lower levels of anxiety related to the disease. This is consistent with findings from various studies conducted on Ghanaian physical education teachers [26], Turkish COVID-19 patients [27], Turkish midwifery students [28], and Qatari and Middle Eastern health care worker [29]. Hence, it is proposed that factors promoting awareness of COVID-19 can contribute to increased levels of consciousness and, consequently, reduce anxiety [30–32]. In our study, we propose that education, as a factor promoting awareness, may have an inverse impact on the anxiety response.

The association between individuals with extensive knowledge about COVID-19 and lower levels of anxiety related to the disease could be attributed to several factors. First, knowledge provides a sense of understanding and control over the situation, reducing uncertainty and fear [33]. When individuals are well-informed about the causes, transmission, prevention, and treatment of COVID-19, they are more likely to feel confident in their ability to protect themselves and others, thereby alleviating anxiety.

Additionally, knowledge about COVID-19 helps individuals differentiate between accurate information and misinformation or rumors. This ability to discern reliable sources and factual information can prevent unnecessary worry and anxiety caused by misleading or exaggerated claims [34].

Some studies, however, report a positive correlation between knowledge and anxiety regarding COVID-19. These studies suggest that individuals who possess awareness of the uncertainties associated with COVID-19 are prone to experiencing heightened levels of anxiety [30, 35, 36]. The increased levels of anxiety could be attributed to the unparalleled fear, apprehension, and nervousness provoked by the COVID-19 pandemic, alongside an increased awareness of the severe repercussions of the virus. There may be several factors contributing to this contradictory finding, including unconsidered moderating effects of other variables and methodological flaws or disparities.

Upon reviewing the existing literature, scant information is available concerning health education interventions specifically targeted towards individuals in quarantine due to COVID-19. In fact, the limited studies available in this area primarily concentrate on the experiences of healthcare workers rather than the general quarantined population. To our knowledge, this is the first randomized controlled trial (RCT) to assess the positive effects of health education on anxiety score among quarantined patients with covid 19.

For instance, a recent uncontrolled trial involving 21 Canadian healthcare workers (HCWs) investigated the efficacy of the RESTORE (Recovering from Extreme Stressors Through Online Resources and E-health) program. The study reported a statistically significant reduction in symptoms of anxiety, depression, and posttraumatic stress disorder [37]. Conversely, another randomized controlled trial (RCT) examining the impact of the PsyCovidApp intervention showed no significant effects on symptoms of depression, anxiety, and stress when compared to a control app among HCWs caring for COVID-19 patients. Interestingly, the effectiveness of the latter app was observed specifically among HCWs who were concurrently receiving psychotherapy or psychotropic medications [38]. But this trial had several limitations.

Furthermore, a longitudinal study was conducted in Tunis by the department of child psychiatry of Mongi Slim. The research team followed 166 children and adolescents with known mental illness via telephone. They concluded that one third of the cohort reported a significant reduction of their anxiety symptoms [39].

As secondary outcomes, neither group in this research showed significant difference of compliance with hygiene measures and adherence to isolation over time. This observation may be attributed to the influence of mass media and widespread awareness campaigns that have targeted the entire population in terms of COVID-19 prevention.

The role of mass media in disseminating information and raising awareness about the importance of hygiene measures and isolation cannot be underestimated [40]. Through various channels such as television, radio, social media, and public health announcements, the general population has been consistently exposed to messages promoting preventive measures against COVID-19. These campaigns have emphasized the significance of practices such as regular handwashing, wearing masks, maintaining social distancing, and adhering to isolation guidelines when necessary.

The extensive reach of these awareness campaigns has likely contributed to a comparable level of knowledge and understanding of hygiene and quarantine measures among individuals from both the intervention and control groups in our study. As a result, there may have been a general consensus and shared understanding among participants regarding the importance of compliance with hygiene measures and adherence to isolation.

The present study had some limitations: All the judgment criteria used were based on the participants’ responses for both the primary endpoint (the HAD scale) or the secondary endpoints (compliance with hygiene measures and with quarantine), which could alter the objectivity of the judgment. However, the use of self-reported scales to measure depression and anxiety is common because of their convenience and low cost. Besides, due to the nature of the intervention, double blinding was not possible. Hence, to minimize the risk of bias the statistical analyzer was blind to his assessment. We also note that our data lack information on confounders like the access to pandemic health education through online enquiry lines or websites of health authorities, socio economic factors and educational level… In addition, the decrease in anxiety and stress could not be conditioned by the content of the educational messages but conditioned by the simple fact of feeling accompanied in the COVID-19 process by a health professional who does observe and guide you in the face of any complications that is why further research evaluating the impact of this accompaniment feeling should be conducted. Another limitation was the exclusive inclusion of subjects with telephones and those who did not require hospitalization, which can alter the generalizability of our results. Lastly, prospective studies often show an important dropout rate, which was the case. Nevertheless, our simple size was large enough to cover this limit. Despite these limitations, the significant differences in psychological profile between the two groups are novel findings providing evidence that health education have an important impact in reducing anxiety levels in subjects with Covid 19 during isolation period.

Such intervention consisting on a psychological support via virtual care (telemedicine, telephone, application…) could be adopted by public health policymakers and physicians while combating the Coronavirus.

## Conclusion

To the best of our knowledge, this is the first study to investigate the impact of health education on mental health symptoms among isolated patients with covid 19. We report that health education has significant impact in reducing anxiety levels of the education group. Therefore, such an intervention could be clinically useful and recommended to alleviate the anxiety of infected patients during an outbreak.

## Electronic supplementary material

Below is the link to the electronic supplementary material.

Supplementary Material 1

## Data Availability

Data are available from the corresponding author upon reasonable request.

## Abbreviations

HAD: Hospital Anxiety and Depression scale
PCR: Polymerase Chain Reaction
D: Day
RCT: Randomized Controlled Trial
HCW: Health Care Workers
DASS-21: Depression, Anxiety and Stress Scale – 21 Items

## References

[1] WHO Coronavirus (COVID-19) Dashboard, https://covid19.who.int (accessed 31. May 2023).

[2] Tunisia. WHO Coronavirus Disease (COVID-19) Dashboard With Vaccination Data, https://covid19.who.int (accessed 31 May 2023).

[3] #HealthyAtHome. https://www.who.int/campaigns/connecting-the-world-to-combat-coronavirus/healthyathome (accessed 31 May 2023).

[4] Dong L, Bouey J (2020). Public Mental Health Crisis during COVID-19 pandemic, China. Emerg Infect Dis.

[5] Brahimi G, Larinouna A, Ait S et al. Etude épidémiologique des patients atteints de Covid- 19 reçus au CHU Béni- Messous du 11 Mars-30 Avril 2020: Résultats préliminaires. 7.

[6] Severely increased generalized. anxiety, but not COVID-19-related fear in individuals with mental illnesses: A population based cross-sectional study in Germany - Eva-Maria Skoda, Alexander Bäuerle, Adam Schweda, Nora Dörrie, Venja Musche, Madeleine Hetkamp, Hannah Kohler, Martin Teufel, Benjamin Weismüller, 2021, https://journals.sagepub.com/doi/10.1177/0020764020960773 (accessed 31 August 2022). 10.1177/0020764020960773 33040668

[7] Giallonardo V, Sampogna G, Del Vecchio V (2020). The impact of Quarantine and physical distancing following COVID-19 on Mental Health: study protocol of a Multicentric Italian Population Trial. Front Psychiatry.

[8] Social Isolation and Anxiety Disorder During the COVID-19. Pandemic and Lockdown in China - ScienceDirect, https://www.sciencedirect.com/science/article/pii/S0165032721006534?via%3Dihub (accessed 31 August 2022). 10.1016/j.jad.2021.06.067 PMC8609130 34256180

[9] Jin Y, Sun T, Zheng P (2021). Mass quarantine and mental health during COVID-19: a meta-analysis. J Affect Disord.

[10] Panda PK, Gupta J, Chowdhury SR (2021). Psychological and behavioral impact of Lockdown and Quarantine Measures for COVID-19 pandemic on children, adolescents and caregivers: a systematic review and Meta-analysis. J Trop Pediatr.

[11] Sepúlveda-Loyola W, Rodríguez-Sánchez I, Pérez-Rodríguez P (2020). Impact of social isolation due to COVID-19 on Health in Older People: Mental and Physical Effects and Recommendations. J Nutr Health Aging.

[12] Lin F-H, Yih DN, Shih F-M (2019). Effect of social support and health education on depression scale scores of chronic stroke patients. Med (Baltim).

[13] Wang J, Yan C, Fu A (2019). A randomized clinical trial of comprehensive education and care program compared to basic care for reducing anxiety and depression and improving quality of life and survival in patients with hepatocellular carcinoma who underwent surgery. Medicine.

[14] Najman N, Kistan K, Novianti I (2020). Relationship on health education against anxiety concerning COVID-19 transmission. ijhs.

[15] Erviana LE, Ismarwati, Isnaeni Y (2022). The Effect of Covid-19 Prevention Education on Public Mothers in reducing anxiety level in the Time Covid-19 pandemic. JHS.

[16] Yang Q, Wu Z, Xie Y (2021). The impact of health education videos on general public’s mental health and behavior during COVID-19. glob health res policy.

[17] Gadi N, Saleh S, Johnson J-A (2022). The impact of the COVID-19 pandemic on the lifestyle and behaviours, mental health and education of students studying healthcare-related courses at a british university. BMC Med Educ.

[18] Aller TB, Kelley HH, Fauth EB (2022). A Non-randomized, quasi-experimental comparison of Effects between an In-person and online delivery of a College Mental Health literacy curriculum. Prev Sci.

[19] Kurki M, Sonja G, Kaisa M (2021). Digital mental health literacy -program for the first-year medical students’ wellbeing: a one group quasi-experimental study. BMC Med Educ.

[20] Akena D, Kiguba R, Muhwezi WW (2021). The effectiveness of a psycho-education intervention on mental health literacy in communities affected by the COVID-19 pandemic-a cluster randomized trial of 24 villages in central Uganda-a research protocol. Trials.

[21] Terkawi AS, Tsang S, AlKahtani GJ (2017). Development and validation of arabic version of the hospital anxiety and Depression Scale. Saudi J Anaesth.

[22] Wang C, Pan R, Wan X (2020). Immediate psychological responses and Associated factors during the initial stage of the 2019 Coronavirus Disease (COVID-19) epidemic among the General Population in China. Int J Environ Res Public Health.

[23] common_mistake_adjusting_sample_size_ct.pdf, https://www.makrocare.com/whitepaper/common_mistake_adjusting_sample_size_ct.pdf (accessed 31 August 2022).

[24] Arafat MY, Zaman S, Hawlader MDH. Telemedicine improves mental health in COVID-19 pandemic. J Glob Health; 11: 03004. 10.7189/jogh.11.03004 PMC8294826 34326984

[25] Hatami H, Deravi N, Danaei B et al. Tele-medicine and improvement of mental health problems in COVID-19 pandemic: A systematic review. *International Journal of Methods in Psychiatric Research*; n/a: e1924. 10.1002/mpr.1924 PMC9349757 35700080

[26] Hagan JE, Quansah F, Anin SK (2022). COVID-19-Related knowledge and anxiety response among Physical Education Teachers during practical In-Person Lessons: Effects of potential moderators. Behav Sci (Basel).

[27] Kef K (2021). COVID-19: the level of knowledge, anxiety and Symptom Presentation. Psychol Res Behav Manag.

[28] Sögüt S, Dolu İ, Cangöl E (2021). The relationship between COVID-19 knowledge levels and anxiety states of midwifery students during the outbreak: a cross-sectional web-based survey. Perspect Psychiatr Care.

[29] Doraiswamy S, Cheema S, Maisonneuve P (2021). Knowledge and anxiety about COVID-19 in the state of Qatar, and the Middle East and North Africa Region-A Cross Sectional Study. Int J Environ Res Public Health.

[30] Wolf MS, Serper M, Opsasnick L (2020). Awareness, attitudes, and actions related to COVID-19 among adults with chronic conditions at the onset of the U.S. outbreak: a cross-sectional survey. Ann Intern Med.

[31] Wang Z-H, Yang H-L, Yang Y-Q (2020). Prevalence of anxiety and depression symptom, and the demands for psychological knowledge and interventions in college students during COVID-19 epidemic: a large cross-sectional study. J Affect Disord.

[32] Yıldırım M, Arslan G, Alkahtani AM (2022). Do fear of COVID-19 and religious coping predict depression, anxiety, and stress among the arab population during health crisis?. Death Stud.

[33] Anderson EC, Carleton RN, Diefenbach M (2019). The relationship between uncertainty and affect. Front Psychol.

[34] Huang Q, Wei L (2022). Explaining education-based difference in systematic processing of COVID-19 information: insights into global recovery from infodemic. Inf Process Manag.

[35] Gong K, Xu Z, Cai Z (2020). Internet hospitals help prevent and control the epidemic of COVID-19 in China: Multicenter user profiling study. J Med Internet Res.

[36] Quansah F, Hagan JE, Ankomah F (2022). Relationship between COVID-19 related knowledge and anxiety among University students: exploring the moderating roles of School Climate and coping strategies. Front Psychol.

[37] Trottier K, Monson CM, Kaysen D (2022). Initial findings on RESTORE for healthcare workers: an internet-delivered intervention for COVID-19-related mental health symptoms. Transl Psychiatry.

[38] Fiol-DeRoque MA, Serrano-Ripoll MJ, Jiménez R (2021). A Mobile phone–based intervention to reduce Mental Health problems in Health Care Workers during the COVID-19 pandemic (PsyCovidApp): Randomized Controlled Trial. JMIR Mhealth Uhealth.

[39] Charfi F, Ben Hamouda A, Bourgou S (2020). Covid-19 and mental disorders in children and adolescents: experience of the child and adolescent psychiatry department of the Mongi Slim Hospital of Tunis. Tunis Med.

[40] Anwar A, Malik M, Raees V et al. Role of Mass Media and Public Health Communications in the COVID-19 Pandemic. *Cureus*; 12: e10453. 10.7759/cureus.10453 PMC7557800 33072461

