# Supplementary material for: The effectiveness of a health education intervention to reduce anxiety in quarantined COVID-19 patients: a randomized controlled trial
Source: BMC Public Health. 2023 Jun 20;23:1188. doi: 10.1186/s12889-023-16104-w (PMC10280925; doi:10.1186/s12889-023-16104-w)
Supplement: Supplementary file 1 — Supplementary Material 1 [file 12889_2023_16104_MOESM1_ESM.docx]

|  | **Total Sample**  **(n= 536)** | **Included for analysis**  **(n= 402)** | **Drop out group**  **(n= 134)** | **p** |
| --- | --- | --- | --- | --- |
| **Age (mean ± SD)** | 41.73 ± 15.53 | 41.12 ± 15.10 | 43.58 ± 16.70 | 0.11 |
|  |  |  |  |  |
| **Gender (n, %)** |  |  |  |  |
| Male | 207 (38.6%) | 152 (37.8%) | 55(41.0%) | 0.50 |
| Female | 329 (61.4%) | 250 (62.2%) | 79(59.0%) |  |
| **Comorbidities (n, %)** |  |  |  |  |
| Diabetes | 46 (8.6%) | 36 [9.0%] | 10 [7.5%] | 0.59 |
| Hypertension | 56 (10.4%) | 40 [10.0%] | 16 [11.9%] | 0.51 |
| Immunodeficiency | 6 (1.1%) | 4 [1.0%] | 2 [1.5 %] | 0.64* |
| Respiratory disease | 28 (5.2%) | 18 [4.5%] | 10 [7.5%] | 0.17 |
|  |  |  |  |  |
| **BMI (mean ±SD)** | 26.98 ± 4.74 | 26.95 ±4.67 | 27.07 ±4.96 |  |
|  |  |  |  |  |
| **Initial HAD-score, Median [IIQ]** | 7[2;16] | 7[2;15] | 7[2;19.25] | 0.32 |
| **Initial anxiety-score, Median [IIQ]** | 3[0;7] | 3[0 ;7] | 2.5[0 ;9] | 0.49 |
| **Initial depression-score, Median [IIQ]** | 4[1;9] | 4[1;9] | 5[1 ;11] | 0.22 |
| **Anxiety (HAD-A) ≥ 8 (n, %)** | 131(24.4%) | 91[22.6%] | 40[29.9%] | 0.09 |
| **Anxiety (HAD-A) ≥ 11 (n, %)** | 74 (13.8%) | 50 (12.4%) | 24 (17.9%) | 0.11 |

**Table: comparison of the dropout group and non-dropout group** **according baseline characteristics.**

* Comparison of percentages with Fisher's exact test
